# Supplementary material for: Are carbon emissions trading and green financial instruments synergistic? -Comprehensive quantitative research based on content analysis
Source: PLoS One. 2024 Mar 7;19(3):e0298601. doi: 10.1371/journal.pone.0298601 (PMC10919601; doi:10.1371/journal.pone.0298601)
Supplement: S1 Data — (ZIP) [file pone.0298601.s001.zip › Data/地级市Z_F工作报告.zip!/░▓╗╒/░÷▓║╩╨/└┤╫╘╣1⁄2╓┌║┼í╛┬φ┐╦╩2╛▌═°í┐_2011.docx]

各位代表：

   现在，我代表市人民政府，向大会作政府工作报告，请予审议，并请市政协委员和其他列席人员提出意见。

   一、攻坚克难争崛起，2010年及“十一五”经济社会发展迈上新台阶

   2010年是巩固经济回升基础、加快跨越发展步伐的重要一年。一年来，全市各族人民在中共蚌埠市委的坚强领导下，坚持以科学发展观为统领，认真贯彻落实国家宏观调控政策，积极应对经济运行中出现的新情况、新问题，团结拼搏，开拓奋进，较好地完成了市十四届人大四次会议确定的目标任务。预计，全年完成地区生产总值600亿元，同比增长13%以上，其中，一、二、三次产业增加值分别增长3.8%、20.3%、9%。财政收入 101.5亿元，增长32.2%，其中，地方收入42.9亿元，增长35.3%。固定资产投资510亿元，增长33%。社会消费品零售总额270亿元，增长18.9%。城镇居民人均可支配收入15370元、农民人均纯收入5295元，分别增长14%、12%。单位生产总值能耗降低4.5%。

   (一)以保持经济平稳较快增长为主线，发展势头进一步向好。

   优先发展工业经济。毫不动摇实施工业强市战略，制定并落实一系列促进工业发展的政策措施，工业主导地位和拉动作用进一步增强。完成工业投资250亿元，增长49%。新增规模以上工业企业200户、销售收入亿元以上企业44户，总数分别达到761户、120户。规模以上工业企业实现增加值237亿元，增长23.5%。加快重点工业项目建设，国瑞特重型数控机床、蚌埠卷烟厂烟叶醇化库等51个项目竣工投产，中建材光电产业基地、柳工起重机底盘、新威(蚌埠)科技产业园等52个项目开工建设。推进开发区扩容升级，新增开发区面积14.16平方公里，新增入区项目208个。高新区升级为国家高新技术产业开发区，怀远马城经济开发区获准筹建省级开发区，固镇台湾工业园获批成立。

   稳步发展农业农村经济。认真落实温家宝总理视察蚌埠重要指示精神，狠抓农业生产，粮食总产达271万吨，增长2.5%，连续七年创历史新高。大力推进农业产业化，农民专业合作社突破400个，新增省级龙头企业10家，江苏雨润、深圳布吉等国家级龙头企业落户我市。扎实开展农田水利基本建设，新增有效灌溉面积4万亩，建设旱涝保收田10万亩。完成成片造林2.6万亩。落实最严格的耕地保护政策，连续12年实现耕地占补平衡。加快土地整治整村推进，实施国家和省级投资土地整理项目58.3万亩。统筹实施38个新农村示范村建设项目。加强劳动技能培训，完成新型农民培训1.8万人。县域经济发展步伐持续加快，生产总值、财政收入等主要经济指标增幅均高于全市平均水平。

   有效提升服务业发展水平。坚持以项目为抓手，宝龙城市广场二期、沃尔玛购物广场投入运营，乐购、来雅百货等知名企业入驻蚌埠，商之都、现代农资机电市场等重大商贸项目基本建成。完善农村市场体系，新建农家店160家，配送中心1家，兑现家电下乡、家电和汽车以旧换新补贴资金1.1亿元。大力发展旅游业，花鼓灯嘉年华落户蚌埠，淮河蚌埠闸创建4A级景区工作加快推进。

   (二)以城市大建设为突破，发展承载力进一步提升。

   全面实施城市大建设。安排重点项目7大类67个，完成投资69.8亿元。拉开城市发展框架，城市南出口改造项目主体完工，西出口改造工程顺利推进，中环线征地拆迁工作已经启动。优化城市路网结构，全年新建改建道路38条，胜利路综合提升改造工程全面开工，大庆路淮河公路桥加快建设，黄山大道一期主体完工。加大重点区域开发力度，高铁新区、滨河西片区、龙子湖西片区综合开发加快推进，河北新区发展步伐加快。推进保障性安居工程建设，陶店新村廉租房竣工交付，喜迎门、陶山安置房和胜利东路公共租赁房项目全面实施，肉厂南村等22个棚户区改造及安置房项目开工建设。

   不断优化生态环境。成功创建省级园林城市，张公岛生态园基本建成，龙子湖北公园二期加快建设，新增城市绿地395公顷。建成19座乡镇垃圾转运站，垃圾渗滤液处理站投入试运行。 

   全力打好节能减排攻坚战，化学需氧量、二氧化硫排放均控制在省下达指标内。

   强化城市综合管理。积极创建第二届省级文明城市。坚持半月推进、逐月点评，推行百分制考核和红黄牌奖惩办法，探索建立城市管理长效机制。加大重点区域、时段监管力度，扎实开展无证无照小饭店、车辆无序停放等专项整治工作，市容环境持续改善。

   (三)以扩大有效投入为抓手，发展后劲进一步增强。

   强力推进项目建设。争取和实施中央新增投资项目106个，落实省以上各类补助资金8.8亿元。全力实施“3461”行动计划，完成投资260亿元，占全市固定资产投资比重超过50%。普乐太阳能电池、华益抗反射膜玻璃等70个项目建成投产，41所依爱电子产业园、合蚌客运专线蚌埠段等131个项目开工建设。开展重大项目谋划和前期工作，新增谋划亿元以上项目117个，总储备规模900亿元。

   有效组织招商引资。创新招商方式，积极开展小分队招商、产业招商和以商招商，组建招商分局5个。大力开展与央企、省企及知名民企对接合作，组织招商引资“百日竞赛”，成功举办第三届中国花鼓灯歌舞节暨蚌埠投资贸易洽谈会。长青集团电动公交车、特步(蚌埠)产业园、中科视讯液晶显示器等125个投资超亿元项目落户我市。全年新引进项目930个，实际引进内资380亿元，利用外资3.1亿美元，分别增长52.6%、16%。加强对外交流与合作，进出口总额突破5亿美元，增长30%。

   不断拓宽融资渠道。积极开展政银企合作交流，全市信贷增量突破50亿元，净投放增长25%。股份制银行引进工作取得重大突破，浦发银行、中信银行在蚌设立分支机构。城投公司20亿元债券顺利发行，佳先化工在天津股权交易所成功挂牌。

   (四)以深化改革创新为动力，发展活力进一步激发。

   加快自主创新步伐。实施产业发展振兴规划，成功创建国家级玻璃新材料和省级汽车零部件、生物等三个高新技术产业化基地。强化创新主体培育，新认定国家高新技术企业21家，新培育省创新型试点企业9家，BB牌滤清器荣获中国驰名商标。加快创新服务平台建设，合工大国家技术转移中心蚌埠分中心、安徽省精细化工(蚌埠)生产力促进中心建成运营，国家特种玻璃及硅材料制品检验中心、蚌埠大学科技园开工建设。深化产学研合作，43个创新产品被认定为国家级重点新产品和省高新技术产品。全年专利授权量900件，增长1倍。

   稳步推进各项改革。企业改制进一步深化。基层医药卫生体制综合改革全面展开。多元化投融资体制初步建立。化解国有粮食企业经营性挂账本金2.45亿元。深化文化体制改革，完成文化市场执法改革等重点任务。开展预算支出绩效评价试点工作，推行县级国库集中支付制度改革。深化农村综合改革，推进农村公益事业建设一事一议财政奖补试点。

   大力发展非公有制经济。新增私营企业1954户、个体工商户10900户。深入推进全民创业，全面开展省级创业型城市创建活动。建成农民工创业园17个。非公有制经济总量达313亿元，占GDP比重52%。

   (五)以保障和改善民生为重点，发展成果进一步共享。

   深入实施民生工程。全面完成33项民生工程，累计投入资金18.3亿元，增长52.5%。市辖区新农合实现市级统筹，完成1所县级妇幼保健院、3所乡镇卫生院、548所村卫生室、36个社区卫生服务中心(站)标准化建设。新建扩建农村敬老院25所。享受义务教育阶段“两免一补”政策78万人次，完成中小学校舍维修改造96.4万平方米。新建农家书屋180个、乡镇综合文化站27个、农民体育健身工程66个。完成村村通公路228公里。建设保障性住房200万平方米，1.76万户家庭享受到廉租住房保障。解决了18万农村人口饮水安全问题。承诺的10件实事全部完成。

   逐步完善社会保障体系。实施更加积极的就业政策，城镇新增就业4.5万人，8.8万人次享受就业补贴。全面落实养老保险省级统筹和失业保险基金市级统筹工作。加快推进新农保试点，发放农村养老金4300万元。增强社会救助保障能力，发放城乡低保金1.97亿元。

   统筹发展社会事业。加强校园环境整治，加大人防技防投入，切实维护校园安全。二中新校区开工建设，蚌埠学院上划省管工作顺利推进。市第三人民医院门急诊楼建成启用，市中医院新院区开工建设。禹墟考古挖掘取得重大成果，固镇垓下大汶口文化遗址入选全国十大考古新发现。举办第五届玉器奇石古玩盆景博览会等文化活动，蚌埠玉器文化城被评为全国文化产业示范基地。圆满完成省第十二届运动会金牌“保二”任务，顺利承办省第六届少数民族传统体育运动会。加大政策扶持和财政投入，保障蔬菜等农副产品供应，加强市场监测监管，千方百计稳定消费价格水平，保障群众基本生活。高度重视安全生产，加强社会治安防控体系建设，加大信访工作力度，社会大局保持稳定。妇女、儿童、青少年、老年人、残疾人权益得到保障。人口和计划生育、国防动员、双拥、统计、人防、审计、外事工作取得新成绩，民族、宗教、司法行政、地方志、气象、地震等工作迈出新步伐。

   (六)以政府效能建设为切入，发展环境进一步优化。

   全面完成政府机构改革，进一步优化政府职能。深化行政审批服务“两集中、两到位”改革，构建基本建设项目联合审批平台。积极贯彻落实行政许可法，开展规范行政处罚自由裁量权试点工作。整合招投标、政府采购、产权交易、土地出让等资源，打造公平、公正、公开的统一的招投标新平台。深入推行政务公开，全文公布清理后保留的316份政府规范性文件。自觉接受人大法律监督和政协民主监督，办理市十四届人大四次会议代表议案7件、建议107件，市政协十二届三次会议提案356件。取消三县车辆通行解放路、朝阳路淮河公路桥收费。加强勤政廉政建设，严格落实《廉政准则》各项规定，大力纠正行业不正之风，严肃查处了一批违纪违法案件，切实整治损害群众利益和破坏投资环境的行为。

   过去一年的发展为“十一五”画上了圆满的句号。“十一五”以来，我们认真落实党中央、国务院和省委、省政府各项决策部署，积极抢抓机遇，有效应对国际金融危机冲击，成功战胜多重自然灾害，努力克服发展中的各种困难，全面完成了“十一五”确定的目标任务。

   过去的五年，是综合实力持续增强的五年。全市地区生产总值年均增长12%，顺利实现翻番。三次产业年均增长4.6%、15.5%和10.8%，二三产业比重较“十五”末提高2.8个百分点，工业化率达40%，经济结构进一步优化提升。财政收入增加1.6倍，其中地方收入增加2.3倍。五年滚动实施“3461”项目628个、累计完成投资近600亿元。社会消费品零售总额、规模以上工业增加值年均分别增长18.1%、19.5%。

   过去的五年，是改革创新深入推进的五年。市属国有企业改制基本完成，政府机构、投融资体制、财政体制等各类改革有序进行，农村综合改革深入实施。成功列入全省合芜蚌自主创新综合试验区，新增各类省级以上科技创新平台60个，创新型企业、高新技术企业、专利申请量等指标跃居全省三甲。对外开放水平不断提高，国电、中建材、中粮、首钢、柳工等一批知名企业落户我市，累计引进资金超1000亿元。开发区开发建设面积较“十五”末增加67.4平方公里。实现进出口总额18.7亿美元，年均增长15%。

   过去的五年，是城乡面貌显著变化的五年。累计完成城市基础设施投资120亿元，市区建成区面积达104平方公里、常住人口105万。新建改建城市道路150条、342公里。城市防洪工程、东海大道景观改造、龙子湖公园一期、淮河蚌埠复线船闸等一批重大基础设施项目建成使用，城市功能更趋完善。棚户区改造取得积极进展，新农村建设扎实推进，城市管理和农村环境整治得到加强，市容环境和村容村貌有较大改善。

   过去的五年，是社会建设全面进步的五年。大力实施民生工程，累计投入资金42.3亿元。不断提高就业和社会保障水平，城乡困难群体得到有效救助。人民生活水平明显提高，城镇居民人均可支配收入和农民人均纯收入年均增长11.6%和13.7%。各类各级教育协调发展，城乡卫生条件持续改善。成功实现全国双拥模范城“四连冠”、全省双拥模范城“六连冠”，荣获全国人民防空先进城市。文化、广播电视、体育等社会事业蓬勃发展。社会主义精神文明、民主法制建设继续加强。

   各位代表，“十一五”时期是我市经济社会发展最快、城乡面貌变化最大、人民群众得实惠最多的时期。成绩令人振奋，经验弥足珍贵。我们必须始终扭住发展第一要务不折腾，紧紧抓住发展这个当前解决蚌埠一切问题的总钥匙，坚定不移地朝着既定的奋斗目标迈进。我们必须坚持调结构、转方式不动摇，在发展中促转变，在转变中谋发展，不断提高经济运行的质量和效益。我们必须紧抓工业强市、扩大投入战略不松劲，以项目为抓手，以项目促发展，围绕关键领域、关键产业寻求重点突破。我们必须勇于争先进位、跨越崛起不停步，立志争创一流，不断实现赶超，用发展的业绩、发展的实力、发展的水平，谋求蚌埠在区域经济和全省格局中应有的地位。我们必须坚持以人为本、改善民生不懈怠，着力解决人民群众最关心、最直接、最现实的利益问题，不断提高人民群众生活水平，始终做到发展成果让群众共享。

   各位代表，五年的成绩来之不易，五年的发展令人鼓舞，五年的成就催人奋进。这是市委正确领导的结果，是全市广大干群共同努力的结果，也是人大代表、政协委员监督支持的结果。在此，我谨代表市人民政府，向全市各族人民，向广大工人、农民、知识分子、干部和解放军指战员、武警官兵、政法干警，向国家和省驻蚌单位，向各民主党派、工商联、各人民团体和其他各界人士，向人大代表、政协委员，向离退休老同志，向在蚌创业的境内外投资者和建设者，向所有关心、支持和参与蚌埠现代化建设的海内外朋友，表示崇高的敬意和衷心的感谢！

   在充分肯定成绩的同时，我们也清醒地看到存在的困难和问题。主要是：经济总量还不够大，发展速度还不够快，经济结构还不够优；缺乏持续带动发展的大企业、大项目，尤其是具有牵动性的重大项目偏少；资金、土地等要素瓶颈制约还没有有效突破；节能减排和不断改善民生的任务艰巨；思想观念还不够解放，加快发展的精力还不够集中，体制机制还不够灵活，政府自身建设还有待进一步加强等。对此，我们一定高度重视，采取有效措施，认真加以解决。

   二、奋发有为谋跨越，大力谱写“十二五”蚌埠发展新篇章

   “十二五”时期，是全面建设小康社会的关键时期，是深化改革开放、加快转变发展方式的攻坚时期，也是我市加快发展、加速崛起，实现跨越赶超、重振蚌埠雄风的重要时期。纵观全局，未来五年我市经济社会发展面临难得的战略机遇。国家继续实施扩大内需政策，加大促进中部地区崛起力度，长三角等发达地区资本和产业加速向中西部转移，我省实施建设合芜蚌自主创新试验区、加快皖北地区发展的重大战略，进一步明确蚌埠中心城市地位，都必将更加有利于发挥我市区位、产业等综合优势，增强发展动力和活力，拓展新的发展空间。同时，经过多年的改革发展，我市综合实力明显增强，经济运行中的积极因素加快集聚，比较优势和后发优势进一步凸显，跨越发展、加速崛起的势头日趋强劲。在此关键阶段，我们必须进一步增强紧迫感、责任感和使命感，坚定信心，抢抓机遇，顺势而为，扎实苦干，在新的起点上推进经济社会更好更快发展。这是我们必须肩负的历史责任，也是全市人民的共同愿望。

   根据市委九届十六次全会通过的《中共蚌埠市委关于制定蚌埠市国民经济和社会发展第十二个五年规划的建议》，“十二五”时期发展的指导思想是：高举中国特色社会主义伟大旗帜，以邓小平理论和“三个代表”重要思想为指导，深入贯彻落实科学发展观，以“科学发展、富民强市”为主题，以加快转变经济发展方式为主线，以升级提速促崛起为总要求，坚持实施工业强市、城乡统筹、开放带动、创新发展、民生优先战略，大力推动工业化、城镇化、农业现代化进程，巩固提升皖北中心城市地位，加快建设宜居宜业宜游特色鲜明的现代化大城市，明显改善人民物质文化生活，为全面建成小康社会奠定坚实基础。

   在市委《建议》的指导下，市政府编制了《蚌埠市国民经济和社会发展第十二个五年规划纲要(草案)》，提出今后五年经济社会发展的主要奋斗目标是：主要经济指标增速高于全省平均水平，地区生产总值年均增长14%，生产总值、财政收入比2010年翻一番，人均生产总值与全省平均水平差距进一步缩小，五年累计完成固定资产投资达到4000亿元以上。产业结构更加优化，服务业增加值翻一番，工业增加值翻一番以上，二三产业增加值占生产总值比重达到86%，工业化率达到50%。自主创新能力显著增强，战略性新兴产业增加值翻两番。利用外资年均增长15%，引进市外资金年均增长20%，进出口总额突破10亿美元。社会消费品零售总额年均增长18%。城镇化率超过50%。城镇居民人均可支配收入和农民人均纯收入年均增长12%，力争五年翻一番。节能减排完成省下达的指标。

   要突出把握好以下五个方面：

   (一)坚持工业强市，加快新型工业化进程。工业是蚌埠的基础所在、优势所在、潜力所在，振兴蚌埠必先振兴工业，必须始终把实施工业强市战略作为奋力崛起的根本途径，着力培育工业发展的内生动力，不断优化工业发展的外部环境，切实增强工业对经济发展的支撑力和带动力。以“培育大企业，发展大产业，做强大工业”为目标，逐步形成龙头企业带动、主导产业引领、园区工业支撑的工业发展新格局。力争五年累计完成工业投资2000亿元，规模以上工业增加值年均增长22%以上，期末工业销售收入超2000亿元。以高端化、规模化、集群化和品牌化为方向，加快提升装备制造及零部件、精细化工、电子信息等优势产业，期末优势产业产值突破1200亿元，成为拉动工业经济增长的主导力量。以核心技术突破和高端人才集聚为支撑，培育壮大光伏、生物、新材料等为重点的战略性新兴产业，期末三大新兴产业产值突破400亿元，成为引领工业增长的先导力量和爆发点。坚持走高端化、特色化和专业化的路子，改造振兴纺织、建材、轻工食品等传统产业，期末传统产业实现产值超过400亿元，稳固工业经济增长的基础。优化产业组织结构，坚持一手抓培育和引进大企业大集团，一手抓支持和服务中小企业加快发展，形成既有“顶天立地”，又有“铺天盖地”，互为依托、竞相发展的生动局面。加快发展现代物流、金融、科技、商务服务等生产性服务业，促进工业与服务业有机融合、互动发展。

   (二)坚持城乡统筹，巩固提升中心城市地位。蚌埠的皖北中心城市定位是历史赋予资源所在、省委省政府战略所在、自身区位优势所在，必须做好这篇大文章，一方面强化自身的实力和魅力，另一方面强化对周边地区的辐射力和影响力，争当皖北崛起增长极和排头兵。挖掘老工业基地和历史积淀潜力，优化交通、科技、教育、卫生、文化、高端商贸和公共服务等资源配置，推动皖北人流、物流、信息流向蚌埠集聚，构建以蚌埠市主城区为核心，怀远、凤阳县城为两翼，沫河口、马城和新马桥为工业卫星城镇的大蚌埠都市区。继续实施“东进、西联、南拓、北跨、中优”的城市空间布局战略，以淮河为轴线，实现拥河发展、分片布局，力争市区建成区面积扩大到140平方公里、市区人口增加到140万人。持续扩大有效投入、多元投入，继续深入实施“3461”行动计划，力争五年滚动实施重点项目超500个。提高土地规划和利用水平，健全规划体系，优化生态环境，提升环境容量，强化项目要素保障。强市必须强县，县区兴则全市兴，必须把加快发展县区经济、增强县区经济活力、尽快提高县区经济在全市经济总量中的贡献度作为重要发展战略。稳步推进城乡一体化，加快城市基础设施向农村延伸，推动城市公共服务向农村覆盖。坚持工业化和城镇化双轮驱动互为支撑，加快三县工业化和城镇化步伐，走出一条新型工业化和集约城镇化的路子来。完善新农村建设规划，形成以三县县城、中心镇、中心村为主体，辐射乡镇和居民点的新农村框架。

   (三)坚持创新发展，增强核心竞争优势。创新是科学发展全面转型的最根本的依靠力量。我市已进入创新累积效应加速释放的关键时期，必须更加积极作为，加快科技和体制创新，推动经济社会发展步入科技引领、创新驱动的轨道。深入推进自主创新综合试验区建设，加快建立比较完善的区域创新体系，为推进发展方式转变、建设创新型蚌埠提供有力支撑。要把科技创新的着力点放到加快新兴产业的发展上来，千方百计培育壮大创新主体，围绕重点产业集群发展，强化产业配套协作，加快培育发展科技型企业。统筹政策支持，全力引进创新型龙头企业，示范和带动战略性新兴产业加速发展。建立健全创新服务体系，组织实施创新平台建设工程，加快建设技术研发平台，积极构建创新服务平台。优化创新发展机制，建立金融保障机制，改革利益分配机制，激发创新动力与活力。推进人才强市战略，实施人力资源开发工程，积极培育创新创业型人才，大力培养高技能人才，努力引进领军型人才，为蚌埠创新发展、跨越发展提供充足的人力保障和智力支持。加快改革攻坚步伐，着力推进行政管理创新，提高政府行政效能，深化国有企业和国有资产管理体制改革，加强现代市场体系建设，加快推进科技、教育、文化、医药卫生、就业和收入分配等社会领域改革。

   (四)坚持开放带动，全面提高对内对外开放水平。伴随国内外产业转移的深入推进和“高铁时代”的加速到来，我市已历史性地站在对外开放的最前沿，必须牢牢把握战略机遇，在更大范围更高平台集聚更多更好的发展要素，形成开放带动发展的强大力量。拓展对外开放的广度和深度，全面加强区域经济合作，加快形成内外联动、互利共赢、稳定高效的开放型经济体系。发挥城市区位、交通、产业等综合优势，在更大范围参与分工和资源配置，积极融入泛长三角合作体系，加强与淮海经济区城市间的交流、合作，主动与合肥、南京经济圈协作互补、融合发展。聚焦我市优势产业和战略性新兴产业，千方百计招大引强，重点引进龙头型、基地型大项目，扩大招商规模，提高引资水平。积极扩大对外经贸合作，努力保持进出口稳定增长，不断提高我市经济外向度。加快构建开放型经济的承载平台，全面推进各类园区建设，以国家级开发区为先、省级开发区为主、工业集中区和专业园区为有益补充，构建层次分明、功能完善的园区经济体系，使园区经济成为我市加快发展的重要增长极。

   (五)坚持民生优先，促进经济社会协调发展。改善民生是加快发展的重要出发点和落脚点，我们必须始终坚持民生优先，从群众最关心最需要的事情做起，把更多的公共资源投向民生领域，实现富民与强市的有机统一。继续实施民生工程，紧紧围绕保障“基本生活、基本养老、基本医疗、基本住房”和困难职工就业再就业、困难家庭子女就学这个现阶段民生问题的核心内容，健全改革体系，加大资金投入，不断提高水平。坚持把增加居民收入作为提高人民生活水平的突破口，大力促进创业和就业，不断强化社会保障，构建人人享有基本保障、待遇水平逐步提高、城乡统筹发展的社会保障机制。按照“加强学前教育、均衡义务教育、规范高中教育、发展职业教育、提升高等教育”的总体要求，统筹发展各类教育事业。大力发展文化事业和文化产业，将文化产业培育成为我市支柱产业，加快实现文化资源大市向文化强市跨越。健全医疗卫生服务体系，提升医疗卫生服务水平。发展群众性体育活动，增强全民健康体质。稳定低生育水平和出生性别比，逐步提高出生人口素质。着力提升生活性服务业，加快商贸服务业集聚发展，积极拓展便民服务业。整合各类社会管理资源，建立政府与社会组织相结合的新型社会管理体制。加强社会治安综合治理，健全覆盖全市的综合防灾减灾体系。加强民主政治和精神文明建设，推进社会主义核心价值体系建设。

   三、全力以赴促发展，努力开创2011年各项工作新局面

   2011年是“十二五”开局之年、蚌埠开埠百年，也是全面开创蚌埠发展新局面、塑造蚌埠新形象的关键一年，做好今年的各项工作意义重大。

   今年政府工作的总体要求是：全面贯彻党的十七大和十七届五中全会及中央和全省经济工作会议精神，以邓小平理论和“三个代表”重要思想为指导，深入贯彻落实科学发展观，以中心城市定位、县域经济发展、工业化城镇化双轮驱动、开放开发引领为主导，着力推动产业发展、提升城市形象、加快项目建设、开展管理创新，更加注重保障和改善民生，保持经济平稳较快发展，促进社会和谐稳定，为顺利实施“十二五”规划开好局、起好步。

   今年经济社会发展的主要预期目标是：地区生产总值增长14%左右，一、二、三产分别增长4%、20%和11.5%，规模以上工业增加值增长23.5%以上；财政收入增长15%；固定资产投资增长30%以上；利用境外资金增长15%，引进市外资金增长30%；社会消费品零售总额增长18%；进出口总额增长15%；城镇居民人均可支配收入增长12%；农民人均纯收入增长12%；城镇登记失业率、人口自然增长率、居民消费价格涨幅、单位生产总值能耗、国家要求削减的主要污染物排放均控制在省下达的目标以内。

   实现上述目标，必须着力抓好以下八项工作：

   (一)坚定不移聚焦产业发展。坚持一手抓龙头、抓集聚，一手抓中小企业、抓全民创业，大力推动产业转型升级，加快构建具有较强竞争力的现代产业体系。力争工业投资增长30%以上，新增销售收入过亿元工业企业30户以上。

   着力培育壮大新兴产业。强化政策集中支持，引进和培育一批领军型龙头企业，加快发展光伏、新材料、生物、装备制造及零部件、精细化工、电子信息等六大产业。推进中建材新光源和电子玻璃、普乐年产100兆瓦非晶硅薄膜太阳能电池、环球药业年产5000万片国家一类新药盐酸安妥沙星、昊方公司空调电磁离合器、天润化工功能高分子材料等项目建设，确保安瑞科压缩机生产基地、中建材光电产业基地等项目开工，争取更多项目列入省“百千工程”。加快中国滤清器产业、省级环保设备制造及柳工起重机制造三个基地建设，提升产业聚集度，为培育装备制造及零部件千亿产业打好基础。围绕支柱产业、新兴产业和重大产品，切实增强自主创新能力。制定实施创新主体培育计划，力争高新技术企业和创新型企业增长20%。加快共性技术研发、成果转化交易、科技资源共建共享等公共服务平台建设，完善创新服务体系，推进产学研结合，新认定高新技术产品和重点新产品30项。加快重大专利技术产业化，专利授权量增长15%以上。支持驻蚌单位在蚌埠加大投入、加快投入、优先发展、更快发展，引导驻蚌单位兴办高新技术企业、科技企业孵化器和科技产业园，发展壮大新兴产业。

   着力改造升级传统产业。加大纺织、建材、轻工及食品等传统产业技术改造力度，大力应用高新技术和信息化技术，积极推广新材料新工艺，加强和改进产品设计，新上一批产业高端项目，增强传统产业新的竞争能力和发展活力。加快推进华润公司啤酒麦芽生产能力扩建，推动安徽中烟1万吨造纸法烟草薄片、特步(蚌埠)产业园、华芳纺织、湖沟煤矿等项目开工建设。发挥老工业基地优势，挖掘人才、技术、品牌等资源，化解历史包袱，激发改制企业活力，加快推进“二次创业”，鼓励创新业、创大业，使改制企业焕发新的生机、实现新的发展、做出新的贡献。

   着力加快发展服务业。大力发展现代物流业，整合本土物流资源，培育3-4家大中型物流企业，引进1-2家国内外知名物流企业，建成4-5家区域性现代物流基地，推进皖北钢材物流大市场、皖北烟草物流配送中心、海通医药物流园建设。鼓励发展法律、会计、中介、会展等服务业，积极探索推进物联网的运用。加快老城商业街区改造步伐，完善新区商业配套，推进淮河路商圈改造提升、张公山商业中心、豪生国际大酒店、金鹰高端商业、金融中心、通成紫都城市综合体、海吉星农产品批发市场等项目建设，开工建设大润发超市和乐购二店，启动开发华夏第一街二期。支持农资、医药等优势企业设立社区、乡镇连锁网点，鼓励大型超市开展“农超对接”。推动房地产市场健康发展。大力发展旅游业，推进涂山、天河、三汊河国家湿地公园、双墩等景区基础设施建设，开工建设东西芦山、沱湖度假村、中华古民居文化园、森禾现代花卉科技产业园等项目。

   着力支持中小企业发展。积极实施高新技术企业“小巨人”计划，鼓励有市场、有潜力的中小企业向专精特新方向发展。支持中小企业依托优势产品，积极引进战略合作者或组团发展。鼓励企业进入资本市场，加快昊方机电、国威滤清器、泰格生物、普乐新能源、天润化工等企业上市进程。深入推进全民创业，通过技能培训、项目推介、基地孵化、资金扶持等多种方式，努力把广大市民的创业愿望变成创业行动。

   着力加强运行调度服务。密切关注宏观形势新变化和新特点，提高监测预警能力。引导企业开发产品、开拓市场，提高市场竞争力。严格落实国家减退税政策，优化企业发展环境，最大限度减轻企业负担。密切关注原材料价格变化，做好煤电油气运等生产要素的协调保障。

   (二)攻坚克难提升城市形象。继续实施城市大建设，坚持扩张外延与提升内涵并重，拉开城市发展大框架，不断改善城乡人居环境，实现承载能力、城市品位双提升。

   加快基础设施建设。提升综合交通配套能力，建成高铁站前广场，完成城市西出口改造，积极推进城市中环线、大庆路淮河公路桥建设，实施城市东出口、北出口改造，开工建设城市外环蚌五高速公路、蚌埠至固镇一级公路。优化城市路网结构，全面完成胜利路综合提升改造、解放路南段、延安路南段、火车站站前道路交通组织等工程，开工建设龙子湖环湖路、东海大道东段。加强市政公用基础设施建设，推进市民广场、综合交通客运站等23项大型公共设施建设。撤销解放路、朝阳路淮河公路桥收费站，实现所有过境车辆免费通行。

   大力实施安居工程。强化拆迁拆违控违工作，突出城市主干道两侧、出入口和项目用地拆迁拆违，完成市区拆迁200万平方米以上。优化安置房建设布局，加快推进钓鱼台棚改、高新区农民新村、戴湖等安置房项目建设，完成100万平方米建设任务。深入推进棚改试点工作，创新棚改模式，新开工建设棚户区改造及安置房项目23个200万平方米。加快保障性住房建设，建成31万平方米廉租住房、13万平方米经济适用房、8万平方米公共租赁房。

   优化城市大环境。把“大建设”与“大绿化”结合起来，巩固扩大省级园林城市创建成果，大力实施城市园林景观建设项目，加快龙子湖北公园二期工程进度，开工建设龙子湖东公园，新建改造一批游园、公园，新增改造绿地65公顷。推进利用日元、世行贷款环境治理及席家沟综合整治工程建设，完善全市污水处理厂配套管网，开工建设城南污水处理厂。推动节能减排技术进步，坚决淘汰落后产能。加强对重点污染企业监管，严格建设项目环境准入，大中型建设项目环评执行率达100%。

   强化城市长效管理。坚持“建管并重、重在管理”的方针，突出抓好占道经营、餐饮卫生、交通秩序、建筑渣土等整治工作，着力解决破墙开店、物业管理、环境保洁等热点、难点问题，实施城市美化亮化工程，力争城市面貌“三年根本改观”。广泛开展形式多样的文明创建活动，提升文明创建水平。

   (三)凝心聚力激活县区经济。加大县区项目、用地、金融、人才等扶持力度，鼓励县区对标追赶，提升县区经济综合竞争力，确保县区主要经济指标增速高于全市平均水平，实现皖北领先、省内进位。

   坚持工业强县不动摇。工业项目偏少、偏小，工业化程度低，是当前三县发展不足、发展不快的症结所在。要紧紧抓住发展工业这个突破口，做到精力向工业集中、资源向工业投入、政策向工业倾斜，千方百计做大做强县域工业经济。围绕农产品加工、不锈钢、纺织服装、机械制造等优势产业，加大政策倾斜和资金扶持力度，每个县培育2至3个特色明显、优势突出、支撑作用和带动能力强的支柱产业和一批骨干龙头企业。抓好信息获取和梳理、登门招商、政策统筹、项目落地准备四个关键环节，引进一些工业大项目，培育一批中小工业企业。

   加快城镇化进程。着力支持县城和扩权强镇试点镇建设，重点扶持马城-常坟、河溜-龙亢、武桥-双忠庙、连城-新马桥组团发展，加快鲍集、沫河口、小蚌埠、长淮卫等重点镇建设。以产业发展、基础设施、公共服务、人口集聚四个关键要素为抓手，创新城镇发展体制机制，加快城镇户籍制度改革，促进各种资源配置向重点乡镇集中。

   大力发展现代农业。认真落实“米袋子”和“菜篮子”工程，搞好农产品生产和流通，确保主要农产品供应充足和价格稳定。稳步提升粮食综合生产能力,力争总产再创新高。推动农业产业化升级，新增市级以上龙头企业10家、市级标准化养殖小区20个。加快农业科技推广步伐，科技入户率达75%以上。全面落实强农惠农政策，积极探索农民承包地流转、农村宅基地确权方面的有效措施，实施新型农民培训，扩大技能型、创业型培训比重，突出发展农产品加工业，多渠道促进农民就业和增收。

   稳步推进新农村建设。全面完成省级新农村示范村项目建设任务，加快农村新型社区建设步伐。大力推进生态家园建设，新建农村户用沼气2000户。着力发展高效林业，完成成片造林5万亩，新增绿色长廊50公里。启动新一轮治淮工程建设，加快实施淮河重点平原洼地治理外资项目和洪泽湖抬高蓄水位影响处理工程。加强农田水利基本建设，新增有效灌溉面积3.5万亩。强力推进农村土地整治工作，建立激励机制，调动乡村和农民积极性，完成21万亩整治任务。

   (四)持之以恒抓好项目投资。牢固树立抓项目就是抓投资、抓投资就是抓发展的观念，强化项目带动，优化投资结构，千方百计扩大有效投入。

   加快“3461”项目建设。健全重大项目市政府直接调度制度，采取项目巡查、现场调度等方式，保障在建项目尽早投产发挥效益、计划新开工项目按期实施。建成华隆消防车基地、中航三鑫太阳能玻璃二期等项目，加快建设年产50万片电容式导电膜玻璃等项目，开工建设微机电系统产业化基地等项目，推进国电蚌埠电厂二期工程等项目前期工作。

   抓好重大项目谋划。强化市级专题谋划小组目标责任制，完善重大项目谋划工作机制。坚持把项目谋划与招商引资结合起来，发挥各类投资主体在项目谋划方面的作用。密切跟踪国家政策导向，突出围绕战略性新兴产业、基础设施、社会事业及民生工程等领域，谋划更多的新项目、大项目、好项目，争取新增谋划项目总储备规模500亿元以上。

   积极拓宽项目融资渠道。紧抓中央扩大内需机遇，强化有关部门责任，加大项目和资金争取力度，各类补助资金比重达到全省平均水平以上。深化银政、银企合作，推进与开行、农发行等政策性银行的贷款对接。加强与商业银行融资合作，确保全年信贷资金增幅高于GDP增幅、高于2010年增幅、高于全省平均增幅。争取再引进1-2家股份制银行在蚌设立分支机构，组建1-2家小额贷款公司。加快推进企业上市融资步伐，争取企业上市取得突破。健全信用担保体系，完成融资性担保公司整顿规范工作。创新风险投资、股权投资、集合债券、质押贷款等金融产品，鼓励支持民间投资建设基础设施、公共事业。加快农村商业银行、村镇银行组建步伐，改善农村金融服务。加强金融生态环境建设，实现经济金融良性互动、和谐发展。

   (五)解放思想加快改革开放。坚持以解放思想为先导，着力实施大开放战略，主攻招商引资头号工程，不失时机地推进各项改革，大力促进对外经贸合作，增添经济发展活力。

   高强度推动招商引资工作。坚持主抓招商引资的决心不动摇，推动招商引资工作再升温、再提速，力争招商引资总量突破600亿元。坚持招大引强，把主攻点放在世界500强、央企、省企、上市公司和知名民企上，着力引进一批投资额度大、科技含量高、产业关联度强的项目。三县四区和高新区、经济开发区力争引进至少一个10亿元以上的项目，并列入考核目标。注重招商选资，破除随机零乱的招商行为，依据项目质量和层次统筹招商政策，真正做到大项目给大政策，好项目给好政策，特殊项目给特殊政策。建立招商项目信息互换交流等机制，健全签约项目责任、通报和协调督办等制度，全面提高项目的开工率、履约率和资金到位率。密切跟踪服务，推进东源环保设备产业基地、奇宏电子(蚌埠)产业基地等重点洽谈项目落地，确保中科视迅液晶显示器、赛远薄钢等合作项目开工建设。

   深化重点领域和关键环节改革。加快县区属国有、集体企业改革步伐。积极推进事业单位改革，加快建立“三定一聘用”管理制度。加强行政事业单位国有资产动态管理，提高资产利用效能。健全财政预算体系，扩大预算支出绩效评价试点范围。深化基层医药卫生体制改革，积极推进公立医院改革。继续深化投融资体制、资源要素价格、农村综合等改革。

   推动外贸增长方式转变。积极扩大对外经贸合作，实施出口品牌战略，引导企业申报国家、省出口名牌，争取更多的出口品牌列入国家、省扶持培育范围。培育壮大丰原集团、丰原生化、昊方机电等重点进出口企业，力争进出口超千万美元企业达到14家以上。加强港口基地建设，充分发挥口岸功能，推动企业就地报关出口。加强国家级精细化工出口创新基地建设，培育省级汽车零部件、玻璃新材料出口基地。积极推动县域外贸发展，力争三县进出口额均突破千万美元。

   (六)统筹兼顾推进园区发展。不失时机扩区升级，主攻基础设施建设和公共服务提升，大力加快园区建设步伐，充分发挥园区工业发展主战场、产业集聚主平台、对外开放主窗口的作用。

   加快高新区开发建设。紧抓高新区晋升国家级的历史机遇，千方百计支持高新区发展。着力突破土地、拆迁、资金三大要素瓶颈，狠抓产业集聚、结构提升两大关键环节，打造推进自主创新示范基地。围绕战略性高新技术产业，在“传统企业高新技术化”和“高新技术产业化”上下功夫，积极引进龙头项目、龙头企业，着力培育集聚上下游企业的产业链。强力推进扩区工作，完善综合服务和基础设施配套功能，拓展发展空间，提升承载能力。

   扩容升级省级开发区。积极探索与发达地区、知名企业合作共建开发区新模式，推动开发区由规模型扩张向内涵型增长转变。加快实施近期扩区方案，拓展园区承载空间，实现省级开发区扩区增容、转型升级。积极创造条件，支持市经济开发区申报国家级开发区。启动马城经济开发区起步区建设，拉开发展框架。完善开发区基础设施，建设一批标准化厂房和公租房，为引进项目配套服务做好准备，争取主动。

   大力发展“板块经济”。加速建设高铁新区，打造集现代商贸、新兴服务业、文化创意产业和生态居住为一体的城市东部综合示范区。充分利用高铁、龙子湖等资源优势，积极开发滨湖新区。加快滨河西片区开发建设，构建西部商贸经济走廊。推进科赛现代工业城、精细化工园、滤清器产业园、生物产业园等各类特色专业园区建设，加速企业集群、产业集聚。

   (七)坚持不懈建设文化强市。经济是城市的肌体，文化是城市的灵魂。蚌埠文化积淀深厚、资源丰富，我们必须坚持社会主义先进文化方向，充分挖掘文化资源，大力发展文化事业和文化产业。

   促进文化事业全面繁荣。加大双墩文化研究力度，继续做好禹墟、垓下等考古发掘工作，加强花鼓灯、泗州戏、五河民歌等非物质文化遗产保护传承。开工建设市档案馆、博物馆新馆等标志性文化设施，建成21个乡镇综合文化站，480个农家书屋。推广农村电影数字放映服务，全年免费放映电影10000场以上。开展蚌埠开埠百年纪念等大型文化活动。

   推进文化产业加快发展。深化文化体制改革，加快8家自收自支经营性文化企事业单位改制。把招引文化项目作为招商引资的重要内容，推进文化与旅游深度融合，加快花鼓灯嘉年华、蚌埠数字动漫基地等文化创意项目建设。做大做强玉器古玩、微雕等艺术品产业，加快南山文化市场、玉器文化城等产业基地建设。积极鼓励和发展非公有制文化企业，培育壮大一批有实力、有活力的文化产业骨干企业。（八）以人为本加强社会建设。坚持把保障和改善民生作为政府工作的出发点和落脚点，切实解决好事关群众切身利益的突出问题，努力让人民群众得到更多实惠。

   始终坚持民生优先。改善民生是政府的应尽之责。我们将继续加大投入力度，不断完善政策措施，调整实施33项民生工程，不遗余力地把顺民意、解民忧、谋民利、保民安、促民和的事情办实办好。增加农村五保户供养、重度残疾人生活和农村义务教育阶段生均公用经费补助，扩大对高校、中职学校家庭经济困难学生资助范围，实施普通高中家庭经济困难学生资助。继续扩大新型农村合作医疗受益面，实现门诊统筹覆盖所有行政村。实施好家电下乡和家电以旧换新政策、农村公路危桥加固改造工程、“一事一议”财政奖补试点等3项新增民生工程。

   完善社会保障体系。加强公共就业服务，实施更加灵活的就业政策，确保城镇新增就业3万人。启动企业人力资源供需对接工程和创业培训工程，完成培训2万人。加强城镇职工医保、居民医保和新农合制度衔接，改进定点医疗机构管理和服务，促进居民就医方便快捷。强化社会救助体系建设，实现城乡居民最低生活保障和农村五保供养应保尽保。

   统筹发展各项事业。大力发展学前教育，进一步提高义务教育均衡发展水平，提升高中教育质量。整合职业教育资源，实施多种形式的校企合作职业教育办学模式。加快推进二中新校区建设，完成蚌埠学院上划工作。深化卫生服务和疾病预防控制体系建设，加快市三院病房大楼、市疾控中心迁建项目建设。深入开展全民健身活动，办好市十三届运动会。努力保持居民消费价格总体水平基本稳定，保障群众基本生活。扩大公积金缴存覆盖面，推行公积金缴存、提取网络化。广泛开展“双拥”活动，加强国防动员和后备力量建设，巩固发展军政军民团结良好局面。切实保障妇女、儿童、青少年、老年人、残疾人权益。推动民族、宗教、外事、气象、地震、地方志事业发展，做好人口和计划生育、统计、审计、人防等工作。

   维护社会和谐稳定。认真落实安全生产责任制，开展安全生产隐患排查和治理，确保安全生产形势稳定。加强食品药品安全监管，打击商业欺诈、非法传销，整顿和规范市场秩序。强化社会治安综合治理，严厉打击各类违法犯罪活动。健全信访工作长效机制，排查化解社会矛盾。完善应急管理机制,增强应对突发公共事件能力，切实维护社会稳定。

   四、提振精神抓落实，树立政府务实高效新形象

   各位代表，做好今年各项工作，实现“十二五”开好局、起好步，需要全市上下共同努力，需要政府自身积极作为。我们要倍加珍惜人民赋予的权力，倍加珍惜难得的干事舞台，倍加珍惜当前的发展机遇，紧盯目标，朝夕勤勉，不怕困难，永不懈怠，努力建设为民、务实、清廉、高效政府。

   坚持执政为民，增强亲和力。加强和改进新形势下群众工作，牢固树立群众观点，创新群众工作方式，提高群众工作能力，切实维护群众合法权益。把政府追求的目标和老百姓的期盼统一起来，健全重大决策专家论证、社会公示和听证制度，完善深入了解民情、充分反映民意、广泛集中民智、切实珍惜民力的决策机制。主动接受社会公众和社会舆论监督，让人民群众知道政府在想什么、做什么，赢得人民群众的充分理解、广泛支持。

   规范行政行为，增强公信力。自觉接受人大法律监督和政协民主监督，努力办好人大代表议案、建议和政协提案，重大事项及时向人大及其常委会报告、与政协协商。认真听取各民主党派、工商联、无党派人士及各人民团体的意见。深入推进法治政府建设，健全行政执法体制，规范行政执法程序。深化“两集中、两到位”改革，认真组织实施基本建设项目联合审批，提高审批服务标准化水平。深入推进政务公开，加快电子政务建设，努力营造办事最快、成本最低、服务最优的政务环境。

   提振精神状态，增强执行力。面对加快发展、加速转型的双重任务，面对人民群众过上更好生活的热切期盼，我们必须始终保持昂扬向上的精气神，始终保持尽心尽力的责任感，始终保持攻坚克难的一股劲，不埋怨，不虚谈，不争论，全身心投入工作。要大兴真抓实干之风，严谨细致，注重实效，让干部用项目说话，让项目为干部说话，努力营造人心思干、思上、思好的生动局面。健全完善目标管理、考核督查、行政问责制度，形成奖优罚劣、跟踪落实和失责必问的工作推进机制。树立“责任比能力更重要”的意识，坚持发展为上、责任为重、落实为要，善于顾全大局，勤于沟通协调，精于创新管理，不在互相抱怨中消磨斗志，不在推诿扯皮中贻误事业，不在消极畏难中错失时机。

   加强廉政建设，增强约束力。全面落实党风廉政建设责任制，严守《廉政准则》，确保干成事、不出事。加大“权、钱、人、地”等重点领域和关键环节监管力度，强化重点建设资金、重大建设项目审计督查，最大限度地减少腐败现象滋生蔓延的土壤和条件。切实珍惜民资民力，把有限的财力物力用在人民群众最迫切需要解决的问题上、用在特殊困难群体的帮助扶持上、用在推进改革发展稳定最关键的事项上。全体政府工作人员特别是领导干部一定要以身作则、勤勉尽责，当好清廉从政的表率，以实际行动取信于民。

   各位代表，加速崛起、重振雄风是全市人民的共同愿望和坚强意志。今天的蚌埠，蓄势待发；明天的蚌埠，更加辉煌。让我们在中共蚌埠市委的正确领导下，高举中国特色社会主义伟大旗帜，深入贯彻落实科学发展观，紧紧依靠和团结全市人民，振奋精神，坚定信心，顽强拼搏，攻坚克难，为圆满完成今年各项任务，为圆满实现我市“十二五”规划目标而努力奋斗！
